# Engaging communities in addressing air quality: a scoping review

**DOI:** 10.1186/s12940-022-00896-2

**Published:** 2022-09-19

**Authors:** Fiona Ward, Hayley J. Lowther-Payne, Emma C. Halliday, Keith Dooley, Neil Joseph, Ruth Livesey, Paul Moran, Simon Kirby, Jane Cloke

**Affiliations:** 1 Division of Health Research, Lancaster University, Lancaster, UK; 2 Applied Health Research Hub (AHRh), University of Central Lancashire (UCLan), Preston, UK; 3 Liverpool City Council, Liverpool, UK; 4 National Institute for Health and Care Research Applied Research Collaboration North West Coast (NIHR ARC NWC), Liverpool, UK; 5 Regenerus, Bootle, UK; 6 Blackburn-With-Darwen Borough Council, Blackburn, UK

**Keywords:** Community engagement, Participation, Air quality, Air pollution, Health inequalities

## Abstract

**Background:**

Exposure to air pollution has a detrimental effect on health and disproportionately affects people living in socio-economically disadvantaged areas. Engaging with communities to identify concerns and solutions could support organisations responsible for air quality control, improve environmental decision-making, and widen understanding of air quality issues associated with health. This scoping review aimed to provide an overview of approaches used to engage communities in addressing air quality and identify the outcomes that have been achieved.

**Methods:**

Searches for studies that described community engagement in air quality activities were conducted across five databases (Academic Search Complete, CABI, GreenFILE, MEDLINE, Web of Science). Data on study characteristics, community engagement approach, and relevant outcomes were extracted. The review process was informed by a multi-stakeholder group with an interest in and experience of community engagement in air quality. Thirty-nine papers from thirty studies were included in the final synthesis.

**Conclusion:**

A range of approaches have been used to engage communities in addressing air quality, most notably air quality monitoring. Positive outcomes included increased awareness, capacity building, and changes to organisational policy and practice. Longer-term projects and further exploration of the impact of community engagement on improving air quality and health are needed as reporting on these outcomes was limited.

**Supplementary Information:**

The online version contains supplementary material available at 10.1186/s12940-022-00896-2.

## Background

Exposure to air pollution has a detrimental effect on health. Outdoor air pollution is estimated to cause 4.2 million premature deaths per year as a result of stroke, heart disease, respiratory disease, and lung cancer [1]. Exposure to air pollution has also been found to be associated with dementia [2], low birthweight [3], and type 2 diabetes [4]. Evidence suggests people living in disadvantaged areas are disproportionately affected, facing a so-called “*triple jeopardy*” where their proximity to sources of air pollution, disproportionate disease burdens, and psychosocial stressors are likely to have a greater negative impact on quality of life [5].

In recent years, there has been increasing attention paid to the participation of communities in identifying and responding to public health issues, such as air pollution. The Marmot Review of health inequalities described this process as “*creating the conditions for individuals to take control of their own lives*” [6]. In the USA, the Office of Environmental Justice recommends involvement of those most effected by poor air quality so that decisions “*best serve*” the interests of the most vulnerable communities [7]. In the UK, the promotion of community engagement is similarly evident with the Department for Environment and Rural Affairs (Defra) expounding the value of local knowledge and interaction with communities to establish the issues in a particular locality and implement solutions appropriate to local circumstances [8]. At a global level, the World Health Organisation (WHO) Helsinki Statement called for governments to include communities in the development, implementation, and monitoring of health considerations in all policies [9].

Community engagement, however, is complex and a term which covers a wide range of approaches to involvement in decision-making and in the planning, design, delivery and governance of initiatives [10, 11]. Engagement approaches vary in the level of community participation, empowerment and control, and consequently have differential impacts on a range of outcomes. Information sharing and consultation exercises, for example, have little impact on health whereas communities who are able to exercise a greater degree of control in an initiative are likely to experience a greater impact [12]. It is also recognised that successful community engagement requires barriers and challenges to be recognised and addressed [13], including factors affecting the ability of organisations to develop and sustain more participatory relationships with communities [14].

Research has shown that community engagement can have positive outcomes for organisations, communities and individuals. Engaged communities working in collaboration with professional or policy stakeholders can increase the system’s understanding of the local context, leading to more culturally appropriate resources and solutions considered to be more responsive to community needs [15]. Pathways through which community engagement may improve health and wellbeing have been identified whereby engagement can have a positive impact on health behaviours and their consequences, either directly through participating in an intervention or via the resulting increase in self-efficacy and/or perceived social support [11]. At a community level, engagement may also engender a greater sense of neighbourhood belonging and improve mental health outcomes [16].

The concept of place is at the heart of community engagement and air quality. Social ties, shared identity or interests in a geographical location or setting are central to definitions of community [17], and “*place-making*” has been described as a way of strengthening connections between people and place [18]. However, the presence of air pollution caused, for example by heavy industry, may also intersect with the ways in which residents identify with their place of residence, as well as perpetuating stigma [19]. The links between health and place have also been widely examined [20], with local social, economic, and political influences identified as important factors in lay perceptions of exposure to air pollution, perceptions of its health impact, and the priority afforded to the issue [21].

Despite the encouragement to engage communities in public health issues and the recognition of the potential for positive outcomes, there has been limited exploration of the range of approaches adopted to engage communities in addressing air quality. This scoping review was conducted to explore the existing literature for possible avenues that communities, researchers and statutory organisations could pursue and identify any directions for further research in this area. Specifically, the review aimed to address the following questions;

1. What approaches have been used to engage communities in air quality?
2. What are the identified facilitators and challenges to engaging communities in air quality?
3. What outcomes have been achieved by engaging communities in air quality?

## Methods

A scoping review, as opposed to a systematic review, aims to capture a broader range of existing literature on a topic, without being limited by study design or quality [22]. This scoping review was designed to collate existing examples of community engagement to addressing air quality and their related outcomes to inform communities, researchers, and statutory organisations wishing to address air quality issues, and to identify gaps for future research. Due to the heterogeneity of studies in this area, a scoping review enabled the synthesis of evidence from relevant studies in a structured and reproducible way. This review was conducted based on existing guidance for undertaking scoping reviews [22, 23], and reported based on the Preferred Reporting Items for Systematic Reviews and Meta-Analyses extension for Scoping Reviews (PRISMA-ScR) checklist [24].

### Search strategy

Preliminary searches were conducted in Web of Science to become familiar with the existing literature on this topic, and to generate and refine the eligibility criteria. A final search strategy was developed and piloted with the assistance of an information specialist. Studies were identified through searching five electronic databases (Academic Search Complete, CABI, GreenFILE, MEDLINE, Web of Science) from their inception to 7^th^ June 2020 using a combination of Medical Subject Headings and keywords. Search terms used across all searches are presented in Table 1. Grey literature was not searched for as this was not feasible with the resources available.

**Table 1:** Search terms

Key concepts	Search terms – combination used across all databases searched
Community	citizen* OR communit* OR neighborhood* OR neighbourhood* OR public* OR resident* OR school* OR stakeholder* OR student* OR taxpayer*
Engagement	action* OR activis* OR consult* OR coproduc* OR co-produc* OR empower* OR engage* OR involve* OR negotiat* OR participat* OR plan* OR research* OR science*
Focus	air pollution OR air quality OR air monitoring

### Eligibility criteria

Eligibility criteria was developed based on the review questions and refined following preliminary searches of the literature. Studies were eligible if they were written in English, undertaken in developed economies and described the active participation of groups and/or individuals of all ages in activities related to air quality in the living environment. Active participation was defined as involving one or more of the following elements associated with research, decision-making and/or actions to address air quality: identifying a problem, priority setting, designing an activity, conducting/delivering, and/or dissemination/sharing learning. The rationale for this definition was to collate examples that go beyond providing information or consultation exercises. The living environment was taken to mean “*any aspect of an individual, group or population’s everyday physical and social environment, excluding the work environment …* [including] *both the socio-economic and psychosocial conditions in which people live*” [25]. The eligibility criteria are outlined in Table 2.

**Table 2:** Eligibility criteria

**Eligibility criteria**	
**Include**	
Country	Developed economies as defined by the United Nations (2020)
Focus/setting	Indoor and/or outdoor air quality in the living environment
Population of interest	Participation of groups and individuals of all ages (including school children)
Studies of interest	No restriction on nature of study design
Language	Written in or translated into English
Intervention/mechanism	Active participation of groups and/or individuals to influence air pollution/quality within the living environment
**Exclude**	
Focus/setting	Wider environmental focus (e.g. health impact assessments, climate change, smoking, nuisance)
Studies of interest	Letters, opinion pieces, review articles, conference abstracts, study protocols and full text not available
Intervention/mechanism	No evidence of active participation (e.g. studies reporting public perceptions, awareness raising, citizen science with no engagement other than data collection)

### Study selection

To increase consistency, two reviewers independently screened approximately 10% of the titles and abstracts of all retrieved citations against the eligibility criteria using Rayyan, a free web-based tool to support collaborative screening [26], and subsequently discussed conflicts in decisions for inclusion or exclusion. As very few conflicts were identified at this stage, the remainder of the titles and abstracts were screened by one reviewer. Full texts of potentially relevant citations were then obtained and independently assessed by two reviewers. Uncertainty or disagreements at any stage were resolved through discussion, and if consensus could not be reached, a third reviewer was consulted and where necessary the wider review group. Reasons for exclusion at the full text screening stage were documented.

### Data charting and synthesis

Data were charted from each of the included papers by one reviewer using a pre-piloted form in MS Excel, all of which was then checked by a second reviewer for completeness and accuracy. The following key data items were taken from each paper: authors, year of publication, country, aim, study design, type and source of air pollution studied, characteristics of the community/study population, approach to engagement, facilitators and challenges to engagement, and reported outcomes associated with engagement. The extent to which health inequalities were considered was also noted during data charting, for example by referencing poor air quality and/or higher rates of respiratory illness in low income communities, environmental justice. As in the practice of scoping reviews, quality assessment was not conducted as studies were not going to be excluded on this basis [23].

To answer the review questions, an inductive approach was used to categorise the approaches used by the studies to engage communities in addressing air quality [27]. As the review aimed to synthesise a potentially broad and diverse area of research, and establish clear links between approaches utilised by the studies, an inductive approach was deemed more appropriate. Starting with the detailed description of the community engagement methods used provided by the authors, studies were grouped into higher-level categories to describe the approach adopted. The categorisation of studies as “*citizen science*” was informed by Den Broeder, Devilee [28], whilst other studies were defined by key words used in their approach to community engagement such as assessment/screening, internship/education, and policy. Detailed summaries of other study characteristics were collated; outcomes were categorised by those observed for individuals, the community, organisations, and those related to air quality and/or health, and the facilitators and challenges to engagement frequently identified by study participants or authors were also summarised. To standardise the categorisation of community engagement approaches and key themes derived from the data items, all included studies were reviewed through discussion until consensus was reached by both reviewers. Decisions were also discussed with the wider review group where necessary.

### Stakeholder involvement

A working group made up of members of the public, environmental health professionals, a local social enterprise, and researchers, all with an interest in or experience of engaging communities in air quality, was formed to support the undertaking of this review. This working group, located within the National Institute for Health and Care Research Applied Research Collaboration North West Coast (NIHR ARC NWC), met on a monthly basis and kept in contact via email to refine the focus of the review, make decisions about the inclusion of studies and types of data to collect, and to interpret key findings. Members were involved in checking the data charting, interpreting the findings, and reviewing this article. An amended version of the GRIPP2 reporting checklist – short-form [29], outlining both public involvement and other stakeholder involvement practices is presented in the supplemental material (Additional file 1).

## Results

**Fig. 1:**
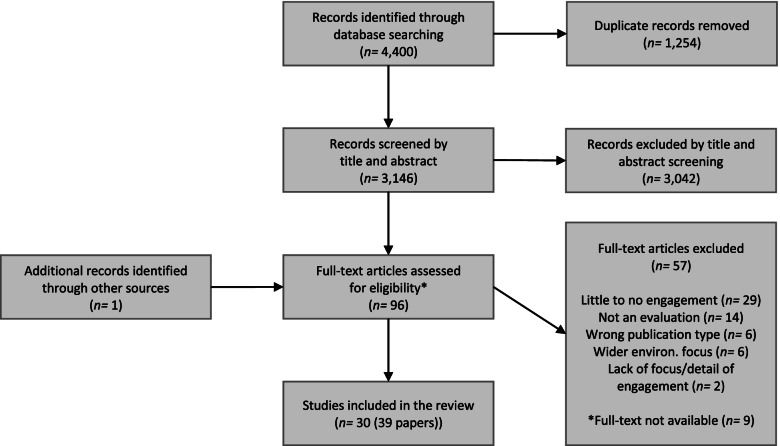
Flow diagram of the study selection process

After the removal of duplicates, the search strategy identified a total of 3,146 citations. Based on screening titles and abstracts, 3,042 citations were excluded. A total of 95 full texts were assessed for eligibility, of which 39 papers were included in the review (Fig. 1).

### Study characteristics

Thirty studies were described across 39 papers (Table 3). Papers were published between 1984 and 2020. Studies were conducted in the USA (*n* = 23), Europe (*n* = 6), and Canada (*n* = 1), most often in urban areas. Most studies focused on engaging communities in activities related to outdoor air pollution (e.g. traffic-related remissions, industrial activity). Study aims were varied, most commonly reported were; to raise individual and/or community awareness of air quality issues, to enable individuals and/or communities to drive action on improving local air quality, and to generate local knowledge of air quality to support environmental decision-making. Studies were often conducted within a community-based participatory research framework [30–41] or action research framework [42–44]; others reported case studies of engagement initiatives [45–48]. Most studies provided little detail about the groups involved in the activities, generally referring to the study population as “*local population*”, “*youth*”, and “*community representatives*”. For studies which provided more information, study populations ranged from 10 to 3,000 members of the community. Evaluations using qualitative methods such as surveys or interviews with relatively small samples were conducted by most studies, usually to generate an understanding of the experience of involvement (e.g. satisfaction) or the impact of engagement (e.g. self-efficacy).

**Table 3:** Overview of included studies

Study title	Study location	Study population	Study aim	Type/source of air pollution	Main approach used	Ref(s)
**Citizen science**						
A Day in the Life	Los Angeles, California, USA	Young people aged 15–17 years (*n* = 18)	Increase environmental health literacy, collect community owned data and promote awareness about air pollution exposure to young people	Indoor and outdoor; multiple	Air quality monitoring	[49]
Air Pollution Assessment in Sheffield	Sheffield, England, UK	Groups of community, environmental, health and business representatives from the local area (*n* = 6)	Explore how focus groups can enable citizens to be involved in local AQ assessment	Outdoor; multiple	Air quality mapping	[50]
AirBeat project	Roxbury, Massachusetts, USA	Children from 3 schools (*n* = 75); community groups and local residents	Describe and analyse the project from the perspective of community-based participants	Outdoor; traffic-related emissions	Air quality monitoring	[35]
Citizen Sense Kit: Just good enough data	Pennsylvania, USA	Local residents (*n* = 30)	Demonstrate that citizen-gathered data can have other uses beyond regulatory comparison and compliance	Outdoor; hydraulic fracking	Air quality monitoring	[51]
Community-empowered AQ Monitoring System	Pittsburgh, Pennsylvania, USA	Community members (*n* = 83)	Explore the use of scientific knowledge in citizen empowerment via information technology	Outdoor; industrial activity	Air quality monitoring	[52]
Creating environmental consciousness in underserved communities	Pittsburgh, Pennsylvania, USA	Local residents—community action team training (*n* = 24); workshop (*n* = 72)	Increase knowledge and understanding of AQ issues within the community and facilitate solutions to the problem	Indoor and outdoor; multiple	Air quality monitoring	[53]
CurieuzeNeuzen Project	Antwerp, Belgium	Local citizens (*n* = 1;840); schools (*n* = 51); hospitals (*n* = 10; companies (*n* = 45); other organisations (*n* = 15)	Examine if participation make citizens more aware of AQ risks and influences their behaviour, and whether the data is acknowledged and used by policy-makers	Outdoor; traffic-related emissions	Air quality monitoring	[54]
Drift Catcher Participatory Air Monitoring Program	Multiple areas across the USA affected by pesticide drift	Pesticide Action Network representatives (*n* = 5); Drift Catcher project leaders (*n* = 2); community members (*n* = 10)	Understand lay involvement in technically complex participatory science projects	Outdoor; pesticide drift	Air quality monitoring	[44]
Improving the Smart Control of Air Pollution in Europe (iSCAPE)	Guildford, England, UK	Local citizens – quiz (*n* = 140); workshop (*n* = 25)	Demonstrate that citizen science can be used to enhance public understanding of AQ by engaging communities and stakeholders	Indoor and outdoor; multiple	Air quality monitoring	[55]
Participatory modelling and local governance of UK AQ	Bristol & York, England, UK	Local citizens	Capture and analyse lay understandings of spatially related environmental issues	Outdoor; multiple	Air quality modelling	[48, 56–58]
Participatory photo-mapping and regional transportation policy	Dane County, Wisconsin, USA	Children aged approx. 11 years (*n* = 9); Adult residents (*n* = 12)	Understand the role of community-based advocacy in advancing public health concerns in transportation planning	Outdoor; traffic-related emissions	Air quality mapping	[45]
Participatory testing and reporting: A pilot project	Worcester, Massachusetts, USA	Residential homes (*n* = 14)	Pilot a participatory testing and reporting programme, aimed at enabling future community-based AQ monitoring	Indoor & outdoor; multiple	Air quality monitoring	[32]
Smart Citizens Lab	Amsterdam, Netherlands	Local citizens	Analyse experiences from using citizen sensing to improve urban AQ monitoring	Outdoor; multiple	Air quality monitoring	[34]
The Imperial County Community Air Monitoring Network	Imperial County, California, USA	Network of community members (*n* = 19); high-school students as interns (*n* = 29)	Educate communities about local AQ, and generate data to find pollution hotspots and trends	Outdoor; multiple	Air quality monitoring, education and training	[41, 59–61]
The Northern California Household Exposure study	Richmond & Bolinas, California, USA	Residential homes (*n* = 50)	Compare an industrial and non-industrial community in an air pollution exposure study	Indoor; emissions from oil refineries	Air quality monitoring	[31]
West Harlem Environmental Action (WE ACT)	Harlem, New York, USA	Young people aged 14–17 years (*n* = 17)	Describe how place-based research and action can contribute to broader policy and change	Outdoor; traffic-related emissions	Air quality monitoring	[36]
Youth Empowerment and Woodsmoke Photovoice study	Washington, USA	Young people aged 13–17 years (*n* = 10)	Understand youth engagement and empowerment in air sampling and photovoice	Indoor; woodsmoke from stoves	Air quality monitoring	[42, 43]
**Environmental/health assessment**						
Community Action Against Asthma	Detroit, Michigan, USA	Children aged 7–11 years diagnosed with moderate to severe asthma (*n* = 3;000)	Assess the effects of AQ on asthma in children, and test interventions to reduce exposure to environmental triggers	Indoor and outdoor; multiple	Health assessment, air quality monitoring	[37, 62, 63]
Environmental Railyard Research Impacting Community Health (ENRRICH)	San Bernardino, California, USA	School community at two comparative schools	Share lessons learned from a partnership assessing respiratory health for children living and attending school near a railyard	Outdoor; railyard	Health assessment, educational theatrical production	[39]
Metal Air Pollution Partnership	Houston, Texas, USA	Local residents – interviews (*n* = 9); focus groups (*n* = 6); surveys (*n* = 375)	Engage communities affected by metal air pollution and build collaboration between relevant stakeholders to develop solutions	Outdoor; metal from recycling plant	Environmental assessment, air quality monitoring	[40]
Wabamun Community Exposure and Health Effects Assessment Programme	Edmonton, Alberta, Canada	Local residents (permanent and seasonal); local community interest groups; aboriginal people	Examine stakeholder relations and risk communication issues, when investigating health effects related to exposure	Outdoor; industrial activity	Health assessment, community surveys	[64]
Wichita Initiative to Renew the Environment (WIRE)	Wichita, Kansas, USA	Community members—WIRE (*n* = 25); discussion groups (*n* = 1;500)	Establish a community-based initiative to identify community environmental concerns	Outdoor; traffic-related emissions	Environmental assessment, educational campaign	[30]
**Education and training**						
Cincinnati Anti-Idling Campaign (CAIC)	Cincinnati, Ohio, USA	School community at local public schools	Develop an anti-idling campaign to decrease children’s exposure to air pollution and reduce asthma morbidity	Outdoor; traffic-related emissions	Educational campaign, air quality monitoring	[33]
Visualising Air Pollution in a Chinese Immigrant Community	Boston Chinatown, Massachusetts, USA	Young people aged 16–17 years (*n* = 9); working age adults (*n* = 21)	Develop and test an intervention to communicate with and empower a community to take action on air pollution	Outdoor; traffic-related emissions	Interactive pollution map, workshop	[65]
**Policy review and development**						
A Case Study of the City of Houston’s Bureau of AQ Control	Houston, Texas, USA	Local residents	Describe Houston’s journey through four levels of public involvement	Indoor and outdoor; multiple	AQ concern reporting system, neighbourhood canvassing	[47]
ClairCity Project	Multiple European countries	Local citizens	Understand the causes of poor AQ and enable citizens to review policies to shape future cities	Outdoor; multiple	Online game, workshop	[66]
Clean Air Task Force	Denver, Colorado, USA	Local citizens	Describe the role of a citizen task force in providing citizens with the opportunity to influence a state AQ implementation plan	Outdoor; multiple	Community task force, workshops	[46]
System dynamics and environmental decisions	Las Vegas, Nevada, USA	Advisory group including community residents (*n* = 30)	Develop policy recommendations to address worsening traffic congestion and regional AQ	Outdoor; traffic-related emissions	Air quality modelling, advisory group	[67]

In the assessment of the extent to which health inequalities were considered, studies referred directly to the engagement activities being conducted with a disadvantaged and/or disproportionately affected group (e.g. low income, ethnic minority). Those undertaken in the USA frequently referred to these as environmental justice communities [38, 40, 45]. Disproportionate exposure to pollution [36, 39, 40, 50, 65], existing vulnerabilities such as age [43, 62], and the marginalisation of certain groups [30, 36, 41, 49] were repeated themes in those placed within the context of health inequalities. Ten studies contained very little reference to health inequalities, with an implicit or explicit suggestion that air pollution was a universal issue within a geographical area [33, 46, 48, 51, 52, 54, 55, 64, 67, 68].

### Approaches to community engagement

#### Citizen science

The most frequent approach adopted was the participation of citizens in air quality monitoring activities; 16 studies featured individuals from local communities involved in the measurement of air quality using scientific equipment or tools. “C*itizen science*”, a term used to describe where citizens contribute to scientific research [28], best encapsulates this type of study. Local residents worked alongside researchers and other stakeholders to identify concerns, influence the focus of the work, define neighbourhood boundaries and select monitoring sites [32, 34–36, 38, 44, 51, 52, 54, 59, 62]. Communities engaged in monitoring received training and were given low-cost sensors or sampling packages to measure local air quality [31, 32, 34–36, 42–44, 49, 51, 53–55, 62]. In some studies, photographs, diaries and stories were also created by residents to supplement the monitoring data [42, 43, 49, 51, 55]. The timescale and extent of the monitoring varied across the studies identified, from mobile sampling over a few days [36], to maintaining monitoring sites for a number of years [41]. Residents also led workshops and community meetings, interpreted data findings, and disseminated information to the wider community and other stakeholders [31, 32, 53]. Fewer studies involved citizens using other scientific methods, including generating “*a lay model of local air quality*” [48, 50, 56–58], creating photo-maps to reflect lived experience of residing in polluted areas [45], and using a smell-reporting system to predict pollution events [68].

#### Environmental and health assessments

For five studies, communities were involved in undertaking environmental or health assessments in collaboration with researchers and statutory organisations. Two environmental assessment studies used participatory methods to identify and prioritise the community’s environmental concerns [30, 40], both recognising this as a knowledge gap and using concerns to develop plans to address these concerns. Studies assessing the health effects of air pollution exposure included respiratory screening of children living near a railyard [39], symptom diaries and lung function assessments for children with asthma [63], and community exposure surveys [64]. For these studies, community members were engaged in multi-stakeholder oversight groups, and contributed to planning, publicity, engaging with the wider community, and dissemination.

#### Education and training

Three studies primarily used education or training programmes to engage the community in air quality activities. Wong, Wu [65] trained high school students to use an interactive pollution map, and supported the young people to teach older adults with English as a second language how to use the map through workshops. To expand a community monitoring network, an internship programme for young people was established by a community-based organisation [60]. An educational programme formed a large part of a community driven campaign to reduce vehicle idling near schools and thus children’s exposure to traffic-related air pollution [33]; videos, assemblies, presentations, signs and factsheets were developed by students and researchers and delivered to parents, students, school staff and bus drivers.

#### Policy development and review

Community engagement in developing and reviewing policies with statutory organisations was a substantial element of four studies [46, 47, 66, 67]. Two reported the inclusion of community representatives on an advisory group to review air quality management policy decisions [46, 67]. Stave [67] conducted a model-building exercise with residents to identify transportation problems and consider policy scenarios to solve these problems. Williams and James [47] described a package of activities to enhance engagement including a public-friendly air quality reporting system, door-to-door canvassing in disadvantaged neighbourhoods, environmental health forums, and citizen-collected evidence of air pollution. The ClairCity project engaged residents and local government in European cities using workshops and an online game to generate citizen-led policy scenarios to improve their cities [66].

The range of outcomes, facilitators and challenges associated with the community engagement approaches used in the included studies are summarised in Table 4. Below, these aspects are described in more detail.

**Table 4:** Community engagement approaches and associated outcomes, facilitators and challenges

Approach	Outcomes (*number of studies*)	Facilitators (*number of studies*)	Challenges (*number of studies*)
**Citizen science** [31, 32, 34–36, 38, 41–44, 49, 51–59, 62, 68]	*Individuals and communities* • Knowledge and awareness of AQ and technical information (e.g. monitoring data) (18) • Capacity building (10) • Empowerment (8) • Confidence and motivation to act (7) • Development of partnerships (6) • Sense of ownership (4) • Self-efficacy (2) • Sense of community (2) • Disappointment or frustration at organisational responses to the project (1)	• Existing partnerships or forums with community-based organisations (6) • Diversity in the community members and research team involved (5) • Existing expertise and experience of communities (5) • Building trusting relationships between those involved (4) • Using a variety of communication mechanisms (4) • Technical support and guidance available (2) • Financial recognition for those involved (2) • Clear plans from the outset (2) • Engaged individuals prior to start of the project (1)	• Use of technical language and communicating scientific material (6) • Capacity of communities (4) • Insufficient resources (4) • AQ sampling issues (4) • Scepticism/lack of trust from communities (1) • Language barriers (1) • Competing priorities (1)
	*Organisations* • New ways of working to address AQ (14) • Local knowledge and experience of AQ (12) • New or revised standards and policies to address AQ (10) • Funding secured for AQ improvements (2) • New ways to work with communities (4)		
	*Air quality (AQ) and health* • Removal or modification of air pollution source (3) • Changes to vehicle idling times (1) • Health protective behaviours (1)		
**Environmental and health assessment** [30, 37, 39, 40, 63, 64]	*Individuals and communities* • Capacity building [5] • Knowledge and awareness of AQ and technical information (e.g. monitoring data) (4) • Sense of community (1) • Development of partnerships (1) • Sense of ownership (1) • Empowerment (1) • Disappointment or frustration at organisational responses to the project (1)	• Using a variety of communication mechanisms (4) • Existing partnerships or forums with community-based organisations (2) • Adapting approach to suit the community (2) • Financial recognition for those involved (2) • Clear plans from the outset (2) • Existing expertise and experience of communities (1) • Building trusting relationships between those involved (1)	• Use of technical language and communicating scientific material (2) • Scepticism or lack of trust from communities (1) • Competing priorities (1) • Personnel changes during the project (1)
	*Organisations* • Local knowledge and experience of AQ (4) • New ways of working with communities (3) • Funding secured for AQ improvements (1) • New ways of working to address AQ (1)		
	*Air quality (AQ) and health* • Access to health services (2) • Identification of undiagnosed asthma (1) • Preliminary data indicated improvements in environmental and health outcomes (1)		
**Education and training** [33, 60, 65]	*Individuals and communities* • Knowledge and awareness of AQ and technical information (e.g. monitoring data) (2) • Capacity building (2) • Confidence and motivation to act (1) • Self-efficacy (1)	• Engaged individuals prior to start of the project (1)	• Use of technical language and communicating scientific material (1) • Language barriers (1) • Insufficient resources (1) • Use of computers for inter-generational learning (1)
	*Organisations* • New ways of working to address AQ (1) • New or revised standards/polices to address AQ (1)		
	*Air quality (AQ) and health* • Changes to vehicle idling times (1) • Health protective behaviours (1)		
**Policy review and development** [46, 47, 66, 67]	*Individuals and communities* • Knowledge and awareness of AQ and technical information (e.g. monitoring data) (2) • Capacity building (2) • Development of partnerships (1) • Empowerment (1) • Disappointment or frustration at organisational responses to the project (1)	• Existing partnerships or forums with community-based organisations (1) • Building trusting relationships between those involved (1) • Existing expertise and experience of communities (1) • Engagement seen as a priority for organisations involved (1) • Adapting approach to suit the community (1) • Diversity in the community members and research team involved (1) • Using a variety of communication mechanisms (1)	• Use of technical language and communicating scientific material (1) • Competing priorities (1) • Scepticism or lack of trust from communities (1) • Language barriers (1) • Use of online methods with disadvantaged communities (1)
	*Organisations* • Local knowledge and experience of AQ (3) • New ways of working with communities (2) • New ways of working to address AQ (1)		

### Facilitators to community engagement

Working with existing community-based organisations that had established links with the local neighbourhood was reported to facilitate community engagement. These organisations were trusted and as such enabled relationship-building between residents, researchers and statutory organisations [37, 39, 40]. Multi-stakeholder steering committees often provided a forum for residents, researchers and organisations with a range of expertise to design and develop activities to address air quality [30, 31, 43]. Trusting and equitable relationships between researchers and residents were seen as a facilitator [35, 36, 39]. Expertise and experience of community members was valued, for example recruiting members of the community with technical skills [35], or local experts to ensure that activities were tailored to residents [53].

Having a range of options for engagement was viewed as important: rather than having a “*one size fits all*” approach, considering each community individually and working in a “*culturally appropriate*” way supported engagement [30, 37, 47]. Effective strategies included using accessible methods [30], and adapting communication to reach different groups [40, 54]. Some studies suggested the integration of community outreach and education from the outset provided a solid foundation for informed community engagement [35, 41, 52]. Diversity in both the community members and the research team was viewed as a critical to success across some studies, particularly in ensuring it was representative of the local population [35, 43, 53, 65]. Lastly, financial recognition for the individuals involved and ensuring that community-based organisations received an appropriate share of the funding was also noted as a facilitator [39, 41].

### Challenges to community engagement

Using technical language and communicating scientific material was the most frequently cited challenge to engaging communities in air quality. Symanski, An Han [40] reported that the use of scientific jargon could decrease community engagement. A focus on the technical aspects limited the role of residents and diminished their sense of ownership [44, 46]. An underlying tension between information being inadequately described or being inaccessible was identified in one study [50]. Communicating scientific information in communities where English was a second language was also acknowledged as a particular challenge [47, 52].

A lack of capacity of community members to be involved in the activities was viewed as a challenge to community engagement; for example limited time available as a result of work or family commitments [32, 40, 43, 67], lack of confidence to be involved in certain elements such as formal presentations [44], and limited access to the internet or equipment [34, 66]. Participatory methods were viewed as more time-consuming and resource-intensive, which could cause capacity issues from an organisational perspective [32, 44]. Additional challenges associated with limited time for researchers or organisations to complete community engagement activities, a lack of access to sufficient resources such as funding and IT experience, and inadequate equipment for residents to sample local air quality, were mentioned by some [34, 36, 43, 54, 65]. Scepticism or a lack of trust limited engagement in some studies; for example, identifying environmental issues being interpreted as criticism of living standards [47], a lack of confidence about the usefulness of air quality models [50], and an inability to overcome existing difficult relationships with statutory organisations [64].

### Outcomes of community engagement

#### Individuals and communities

Increased knowledge and understanding of air pollution, its causes and the associated health outcomes was identified across most studies: this was often highlighted as a prerequisite for engagement and studies frequently described an initial stage of outreach activities to share information with residents. Some studies highlighted an increased understanding of technical information and monitoring data [35, 49, 53, 67], whilst others reported engagement increased awareness of the cumulative impacts of air pollution, the burden of ill health associated with social vulnerability, and the injustice of exposure [38, 42, 53]. In addition, studies suggested a consequence of this increased awareness would likely be personal behaviour change to reduce exposure [54, 59, 67], however this effect was not formally measured.

Many studies outlined how residents had developed skills and competencies in, for example, conducting research [30, 62], using monitoring equipment [34, 59], action planning [59], leadership [35, 40], public speaking and dissemination [30, 53, 60], and advocacy [31, 36, 62]. Participation in air quality activities was found to have enhanced individual and collective confidence and motivation to act [31, 42–44, 49, 52, 64, 68], built a sense of community [37, 52], and increased self-efficacy [38, 52, 65, 66]. Community engagement in some cases enabled the development of new connections [41, 59, 63, 65], and partnerships between the community and organisations [37, 47, 53]. Community participation in air quality activities was found to elicit a sense of ownership, particularly in studies whereby residents has contributed to decision-making or collected their own data [30, 35, 38, 68]. Individual or community empowerment was frequently described in the studies, a potential product of these linked competencies [35, 40, 42, 43, 52, 55]. Some studies described the achievement of these linked outcomes as increased environmental health literacy [40, 49, 60].

For a small number of studies, communities reported experiencing disappointment or frustration at statutory organisations’ responses [37, 50, 68]. In two cases, the processes and/or inclination did not exist for lay knowledge and citizen-collected data to be integrated into the work of statutory organisations [46, 51]. One study identified tensions with local media suggesting that the public were being misled about the extent of the links between air pollution and health [35], whilst another reported that the use of personal air monitors had the potential to prioritise individual behavioural responses, transferring the responsibility for action from the producers of emissions to vulnerable populations [49].

#### Organisations

Engaging the community often led to changes for statutory organisations. Additional funding or the maintenance of funding to address air pollution was secured as a result of the engagement activities in two studies [41, 43]. New or revised policies emerged from some studies, such as the development of an air protection policy [41], formation of greener zones [38], and policy changes on vehicle idling and industrial site regulation [33, 36, 68]. Hayes, King [66] focused on a citizen-led review of existing policies and showcased new methods for policy co-creation with citizens. A wide range of changes to practice were described including implementing cleaner fuel for public transport [36], a 24-h call system to respond to community concerns [40], new monitoring sites [41, 51], and changes to school environments [39]. In some studies, residents or community-based organisations continued to contribute to decision-making on air quality through ongoing engagement activities, including advisory and policy-making boards [37], environmental health forums [47], volunteering schemes [40], and embedding participatory modelling into usual practice [57]. Having engaged with local residents, additional information was now available to statutory organisations. An improved understanding of pollution sources was reported [59, 61, 62], in addition to increased awareness of local community concerns and the challenges they face [30, 39, 52, 64].

#### Air quality and health

Very few studies evaluated the impact of their community engagement activities on local air quality and/or health. Two studies measured environmental outcomes: preliminary data indicated improvements in one study [63], and another saw a reduction in bus and car idling times, their simulation suggesting improved air quality [33]. Studies frequently concluded that if specified changes in policy and practice were successfully implemented, air quality in and around specific buildings such as schools, or in neighbourhoods close to sources of industrial pollution would improve [35, 36, 39, 42, 44, 52, 66]. As with the environmental impacts, there was an assumption, rather than measurement, of health improvement through the adoption of new practices and policies (such as the use of an air pollution alert system) which meant that residents were now equipped with information to protect their own health [41, 65].

## Discussion

This scoping review aimed to explore approaches used to engage communities in efforts to address air quality. Disproportionate exposure to air pollution was frequently considered, particularly in studies conducted in the USA, which were often located within “*environmental justice*” communities. The approaches used by studies were varied, but have been usefully categorised into citizen science, environmental and health assessment, education and training, and policy development and review. The community engagement initiatives reported a number of positive outcomes for individuals and communities, including increased awareness, enhanced self-efficacy, community connectivity, and for the organisations involved, including access to local intelligence, and development of new policies and practices. However, limited evidence existed on the extent to which engagement led to changes in health or environmental outcomes for individuals and local populations, as these outcomes were largely not measured in studies.

### Contributions to existing research

Although air quality monitoring initiatives were most prominent in this review, a range of other approaches to engage communities in air quality were also evident and reported positive outcomes. Including studies which utilised environmental and health assessments, education and training, and policy development and review, the findings add to the review of community participation in air quality monitoring studies conducted in the USA [69], by offering illustrative examples for communities, researchers and organisations wanting to collaborate in this area, and also suggesting alternatives where monitoring may not be appropriate. Case study examples of community action influencing policy changes reported in this review [33, 35, 36, 44], reflect other studies that have demonstrated the potential for participatory approaches to result in policy change in addressing environmental concerns [70, 71]. A case study of industrialised hog production cited by Whitehead, Pennington [25], for example, highlights how a community health and environmental partnership involving the local community and researchers led to heightened attention of the health hazards associated with hog production particularly among African American communities and was used to challenge the health damaging effects of industrial production. Israel, Schulz [72] and Wine, Ambrose [73] have highlighted how community-based participatory approaches are more likely to attend to issues of power and equitable relationships compared to other community engagement approaches, which in itself could be empowering for communities and have benefits for wellbeing and trust.

Whilst many of the facilitators (e.g. using existing partnerships, adapting methods to the community, building relationships) and challenges (e.g. limited community capacity, scepticism and trust, insufficient resources) to community engagement identified were consistent with findings from other reviews [13, 72] and were not specific to air quality studies; others were more salient and require particular consideration such as the use of technical or scientific language, and internet access needed by participants to, for example, upload and view monitoring data. Furthermore, while positive organisational outcomes and changes to policy and practice were identified in this review, little information was provided about the capacity needed to facilitate community engagement and maintain ongoing relationships. Considerations included not only appropriately skilled staff but also an organisational ethos and culture that is positive about community engagement and systems in place to support this [14]. Additional resource in air quality initiatives may be required, for example, to obtain and maintain equipment [32].

The importance of considerations of place and health inequalities in addressing air quality was evident across the studies. Whilst there is impetus to reduce air pollution risks for all affected populations, communities living in more disadvantaged areas are at a disproportionate risk of inequities in air quality and health outcomes [5]. Engaging disadvantaged communities and locating air quality issues and solutions within the framework of the wider social and economic determinants of health may achieve health gains, both in terms of reduced health risk and health inequalities [5]. Whilst Noël, Vanroelen [21] found evidence that air pollution could be “*crowded out*” by other personal and urgent issues, the learning from this review is that, from the studies in environmental justice communities in particular, successful collaborations can be built in vulnerable communities. Using air quality monitoring and/or other activities, key components of these approaches included establishing public concerns, community capacity building, adapting strategies to meet the needs of the community, and having equitable partnerships amongst those involved [31, 35–38, 53]. A recent interpretative synthesis also highlighted the greater likelihood of environmental justice communities achieving “*structural change*” (e.g. impacting the wider determinants of health) when partnerships were long-term, project design included decision-makers and policy goals, and community members held leadership roles [74]. In addition, previous reviews have placed an emphasis on studies of community engagement addressing health at a more individual level (e.g. smoking cessation) or with groups that share a social or cultural identity [11, 72]. For studies included in this review, communities have a specific shared identity based on place and this was grounded in their lived experience of poor air quality, and reinforces the need to implement place-based approaches to facilitate action on air quality in collaboration with local communities.

### Limitations of the evidence base

Due to the varied nature of the literature and lack of detailed reporting, drawing on comparisons between community engagement approaches, the communities involved, and assessing effectiveness was challenging. Previous reviews have found similar limitations in the evidence base and challenges with associating outcomes with a specific approach to community engagement and the context in which it was conducted in [11, 75, 76]. The summary presented in Table 4 characterises the community engagement approaches and the variety of associated outcomes, facilitators and challenges found in this review. However, some caution should be noted in its interpretation because of the heterogeneity of the evidence base.

Related to this, a clear gap found consistently across the community engagement literature was in relation to outcomes and the wider determinants of health. Whilst positive impacts for engaged individuals, communities and organisations were reported in many studies, few theorised whether engagement contributed to changes which reduced air pollution or improved health, or measured this in their research. These findings are in line with other reviews [11, 75, 76], which identified short-term outcomes for community engagement (e.g. self-efficacy, empowerment, policy change), but found studies collected or reported insufficient data to test the effects of community engagement on longer-term health outcomes.

A paradox therefore remains in that while community engagement approaches demonstrate promise in tackling environmental justice, the availability of robust evidence is still limited. While in part this is down to a lack of theorisation or measurement, it’s “*grassroots*” nature can also make some community engagement less amenable to more traditional evaluation designs [77]. In this vein, Israel, Schulz [72] highlighted in a review of community-based participatory research that the ability to secure research (or other) funding for such initiatives stems from “*a challenge of selling a process without completely specifying all the outcomes beforehand, often troubling for researchers, health professionals, and community members, as well as funders*”. A review undertaken several years later which researched the effectiveness of initiatives to strengthen community control in the living environment, suggests this research gap still remains, with many examples of practice also still remain located in descriptive case studies or unpublished sources making it difficult to judge their “*comprehensive or quality*” [78].

### Implications for future research and practice

Within recent years, there has been increased research and policy attention to air pollution and its impact of health [79, 80], with recommendations that communities should be engaged in identifying issues and solutions to poor air quality in their neighbourhoods [7–9]. The findings of this scoping review can be used to inform the future development and implementation of these approaches. Firstly, in the context of addressing air quality, successful community engagement approaches appear to require strategies that enable effective collaboration between communities, researchers and organisations (e.g. multi-stakeholder forums), offer opportunities to embed lived experience and local knowledge of poor air quality (e.g. monitoring, mapping), and promote a wider “*outward gaze*” [81] to the political and social structures that influence addressing action on this public health issue. Secondly, developing a reporting checklist, similar to the Guidance for Reporting Involvement of Patients and Public (GRIPP2) [29], or the Template for Intervention Description and Replication (TIDieR) [82], may improve future reporting of community engagement initiatives and support attempts to compare and contrast approaches and their effectiveness. As far as the authors are aware, no such checklist exists. With enhanced reporting, communities, researchers, and organisations, could identify what form of community engagement may work best in their specific context and replicate the approach to achieve their desired outcomes. Lastly, although establishing a causal relationship between community engagement and reduced air pollution or improved health is difficult [79], future research should consider from the outset how the pathway from the engagement activity to any subsequent impact on air quality and health can be elucidated. In this respect, evaluation methods adopting systems approaches can help in elucidating the complexities in environmental settings, by enabling inter-related changes and pathways to health to be captured [83]. For example, air quality measures that have potential to benefit health directly (e.g. respiratory conditions) could also result in other impacts that are health enhancing (e.g. changes to traffic flows leading to improved road safety or increased physical activity and social engagement within a community).

### Strengths and limitations of this review

A strength of this scoping review was the embedding of stakeholder and public involvement throughout the review process, enabling the review questions, data extraction, interpretation and writing to be shaped by a group of individuals with a range of personal and professional perspectives. The reflections on the impact of this involvement are outlined in the supplemental material (Table S1). As the aim of this review was to capture a breadth rather than a specific standard of evidence, issues associated with quality appraisal were not addressed. It was however conducted in line with existing guidelines for scoping reviews [22, 23]. This review was not intended to be exhaustive or comprehensive and some relevant examples may have been missed. For pragmatic reasons, including limited time and resources, additional citation tracking and grey literature searches were not conducted, and as such may have limited the extent to which further examples of community engagement initiatives in air quality were identified. This review was also limited to studies conducted in developed economies and to studies concerned with indoor and/or outdoor air quality in the living environment; additional insight of community engagement initiatives in other contexts may be beneficial.

## Conclusion

This scoping review summarises the variety of approaches that have been used to engage communities in addressing air quality, highlighting some of the facilitators, challenges and possibilities available to communities, researchers and statutory organisations wishing to undertake work in this area. The findings suggest that positive individual, community and organisational outcomes can be achieved through multi-stakeholder collaborations working with researchers. The limited evidence available on the impact of community engagement on improving air quality and health, and consequently addressing associated health inequalities has identified a need for future studies to explore and clarify this pathway.

## Supplementary Information

**Additional file 1: Table S1. **GRIPP2 reporting checklist – short form (amended)

## Data Availability

All data generated and analysed in this review are included in this article and its supplementary information files.

## Abbreviations

ARC NWC: Applied Research Collaboration North West Coast
GRIPP: Guidance for Reporting Involvement of Patient and Public
NIHR: National Institute for Health and Care Research
PRISMA-ScR: Preferred Reporting Items for Systematic Reviews and Meta-Analyses extension for Scoping Reviews
UK: United Kingdom
USA: United States of America
WHO: World Health Organisation
