# Supplementary material for: Engaging communities in addressing air quality: a scoping review
Source: Environ Health. 2022 Sep 19;21:89. doi: 10.1186/s12940-022-00896-2 (PMC9484248; doi:10.1186/s12940-022-00896-2)
Supplement: Supplementary file 1 — Additional file 1: Table S1. GRIPP2 reporting checklist – short form (amended) [file 12940_2022_896_MOESM1_ESM.docx]

**ADDITIONAL FILE 1**

**Table S1.** GRIPP2 reporting checklist – short form (amended)

| **Section and topic** | **Details** |
| --- | --- |
| 1: Aim of the stakeholder involvement (SI) | To involve wider stakeholders (including organisations and members of the public) as research partners throughout the scoping review process. |
| 2: Methods used for SI | Two environmental protection professionals, a representative from a local social enterprise and two public contributors were recruited through the NIHR ARC NWC to a working group to support the review process. Members of the working group all had interest in or experience of engaging communities in air quality. The working group met regularly and at key points in the study to discuss the review and communicated via email throughout the review process. |
| 3: Results of the SI | The working group provided a strong and varied stakeholder perspective, contributing to the review in several ways, including;   - refining the focus of the review questions - reviewing summaries of the papers identified and using their experience to highlight whether it constituted ‘active participation’ of communities - informing what data would be of interest to be collected from the papers from a public and professional perspective (e.g. outcomes for individuals and the community as a whole, impact on health and air quality) - checking some of the data charting conducted by researchers to ensure completeness and accuracy of this process - interpreting the findings, highlighting the themes that were most important from a public and organisational perspective (e.g. collaborative working, use of technical language, challenges faced by statutory organisations) - and contributing to the edits of the review write-up and suggesting ways to disseminate the findings. |
| 4: Discussion and conclusions of the SI | Feedback was sought from the working group members about their role in conducting the review. Comments suggested that they valued being involved from the early stages of the review process and that this reinforced the co-design approach. As part of the working group, they felt able to influence the scoping review through their participation in meetings, active discussion on the review process, and contributing to key stages such as inclusion decisions, data charting and interpreting the findings. Their specialised knowledge and experience of community engagement initiatives and roles within statutory organisations ensured that the review addressed questions pertinent to communities, added a different perspective to working group discussions, and supported tailored dissemination of the findings. Group members also reported that through their involvement they had learnt more about, for example, approaches to community engagement, citizen science and challenges in addressing air pollution. |
| 5: Reflections/critical perspective of the SI | Although the impact of SI in this scoping review is difficult to measure robustly, the feedback that has been received suggests that the contributions of the working group members influenced the review process and validated many of the findings related to positive outcomes observed for individuals and communities as a result of being engaged in air quality activities. A challenge to SI in this review was the accessibility of working group meetings as due to the COVID-19 pandemic, meetings were held virtually; this was not always an ideal format for collaboration. Further engagement with a wider group of individuals may have also enhanced the SI in this review (e.g. children and young people, people living and/or working in another geographical area). |
